# Mechanisms of Immune Escape and Resistance to Checkpoint Inhibitor Therapies in Mismatch Repair Deficient Metastatic Colorectal Cancers

**DOI:** 10.3390/cancers13112638

**Published:** 2021-05-27

**Authors:** Vito Amodio, Gianluca Mauri, Nicole M. Reilly, Andrea Sartore-Bianchi, Salvatore Siena, Alberto Bardelli, Giovanni Germano

**Affiliations:** 1 Candiolo Cancer Institute, FPO-IRCCS, 10060 Candiolo, TO, Italy; vito.amodio@unito.it (V.A.); nicolemegan.reilly@unito.it (N.M.R.); 2 Department of Oncology, University of Torino, 10060 Candiolo, TO, Italy; 3 IFOM, The FIRC Institute of Molecular Oncology, 20139 Milan, MI, Italy; gianluca.mauri@unimi.it; 4 Niguarda Cancer Center, Grande Ospedale Metropolitano Niguarda, 20162 Milan, MI, Italy; andrea.sartorebianchi@unimi.it (A.S.-B.); salvatore.siena@unimi.it (S.S.); 5 Dipartimento di Oncologia ed Emato-Oncologia, Università degli Studi di Milano (La Statale), 20162 Milan, MI, Italy

**Keywords:** colorectal cancer, mismatch repair deficiency, MSI, microsatellite instability, immune escape, immune surveillance, immune evasion, immune checkpoint inhibitors

## Abstract

**Simple Summary:**

A subset of colorectal cancers (CRCs) is characterized by a mismatch repair deficiency that is frequently associated with microsatellite instability (MSI). The compromised DNA repair machinery leads to the accumulation of tumor neoantigens affecting the sensitivity of MSI metastatic CRC to immune checkpoint inhibitors (CPIs), both upfront and in later lines of treatment. However, up to 30% of MSI CRCs exhibit primary resistance to frontline immune based therapy, and an additional subset develops acquired resistance. Here, we first discuss the clinical and molecular features of MSI CRCs and then we review how the loss of antigenicity, immunogenicity, and a hostile tumor microenvironment could influence primary and acquired resistance to CPIs. Finally, we describe strategies to improve the outcome of MSI CRC patients upon CPI treatment.

**Abstract:**

Immune checkpoint inhibitors (CPIs) represent an effective therapeutic strategy for several different types of solid tumors and are remarkably effective in mismatch repair deficient (MMRd) tumors, including colorectal cancer (CRC). The prevalent view is that the elevated and dynamic neoantigen burden associated with the mutator phenotype of MMRd fosters enhanced immune surveillance of these cancers. In addition, recent findings suggest that MMRd tumors have increased cytosolic DNA, which triggers the cGAS STING pathway, leading to interferon-mediated immune response. Unfortunately, approximately 30% of MMRd CRC exhibit primary resistance to CPIs, while a substantial fraction of tumors acquires resistance after an initial benefit. Profiling of clinical samples and preclinical studies suggests that alterations in the Wnt and the JAK-STAT signaling pathways are associated with refractoriness to CPIs. Intriguingly, mutations in the antigen presentation machinery, such as loss of MHC or Beta-2 microglobulin (B2M), are implicated in initial immune evasion but do not impair response to CPIs. In this review, we outline how understanding the mechanistic basis of immune evasion and CPI resistance in MMRd CRC provides the rationale for innovative strategies to increase the subset of patients benefiting from CPIs.

## 1. Introduction

The introduction of checkpoint inhibitors (CPIs) has radically changed therapeutic strategies used for many types of solid tumors, such as melanoma, breast, cutaneous squamous, head and neck, renal, urothelial, gynecologic, and thoracic cancers [1,2,3,4,5,6,7,8,9,10,11,12,13,14,15]. Intriguingly, in mismatch repair deficient (MMRd) malignancies, CPIs have been shown to be particularly effective, thus leading to the first tissue agnostic approval of pembrolizumab based on microsatellite instability (MSI) molecular status only [16,17].

Colorectal cancer (CRC) represents the third most common type of tumor and accounts for more than one third of cancer-related deaths in both genders [18]. Considering all stages of CRC, overall survival (OS) in patients is around 60% at 5 years from initial diagnosis, but survival rates dramatically decrease to 15% in patients with stage IV metastatic CRC (mCRC) [19,20]. In patients with liver metastases, surgical resection within a multidisciplinary therapeutic strategy should always be considered, as it is the only treatment that can provide the possibility of prolonged survival, or even cure [21]. In particular, over the last few decades, survival of patients with liver-limited disease has improved dramatically due to the extension of surgical indications and the development of innovative surgical procedures [21]. CRCs can be biologically classified into two subgroups according to the microsatellite stability classification: a) mismatch repair proficient (MMRp) tumors are defined as microsatellite stable tumors (MSS), since the length of microsatellites is stable over time; b) mismatch repair deficient (MMRd) tumors may generate a microsatellite unstable (MSI) phenotype in which the length of the microsatellite regions changes during cell division [22]. MSS tumors represent the vast majority of CRCs, whereas MSI are up to 15% in early stages (I-III) and only 5% in stage IV [23,24]. These two subgroups of CRC reflect two diseases with different etiologies, clinic-pathological features, and outcomes to standard cytotoxic combinations or CPIs [25]. MSI CRCs are more frequently located in the right colon, are poorly differentiated, and have mucinous features [26] Around 3% of MSI CRCs arise in the context of Lynch syndrome due to germline mutations in mismatch repair (MMR) genes [26]. Alternatively, around 12% of MSI CRCs are sporadic due to somatic hypermethylation of CpG islands, known as the CpG island methylator phenotype (CIMP), often surrounding the promoter region of *MLH1* [27]. Furthermore, *BRAF* mutations are significantly more common in MSI rather than in MSS CRC (34% vs. 6% of cases) [28]. MSI metastatic CRC are characterized by poorer prognosis, and they are usually resistant to common cytotoxic and targeted agents [24,29,30]. Interestingly, while MSS mCRCs exhibit primary resistance to CPIs, MSI mCRC are greatly sensitive to CPIs [16,31,32].

In this review, we first describe the main molecular features of MSI mCRC, then focus on genetic and non-genetic mechanisms of immune-evasion and resistance to CPIs. Finally, we provide perspectives on potential strategies aiming to prevent or overcome the occurrence of these resistance mechanisms to CPIs during clinical treatment of mCRC.

### 1.1. The Role of the Gut Microbiota in the Initiation and Progression of MSI CRC

In the last decades, several studies shed light on how gut microbiota influences cancer initiation and development, and studies have found overwhelming evidence that the microbiome influences disease progression and clinical outcomes. The microbiome is composed of up of 100 trillion different bacteria, viruses, fungi, and protozoa, and the presence of a large and diverse microbiota population in the gut has a role in the maintenance of the physiological gastro-intestinal activity, including vitamin metabolism, prevention from pathogen infections, and regulation of epithelial homeostasis [33,34]. Finally, the microbiota regulates the maturation of the mucosal immune system, while the pathogenic portion may cause immune dysfunction and disease development [35,36]. In cancer, microbiome perturbation is associated with tumorigenesis and progression, affecting several cancer evolutionary processes [37,38]. Particularly, the association between CRC and microbial composition was first reported in 1975 in a study that showed how tumor incidence increased in germ-free rats upon exposure to n-methyl-n’-nitro-n-nitrosoguanidine [39]. Now, it is well known that the imbalance (or dysbiosis) of gut microbiota, with the expansion or depletion of certain species, is the cause of several disease including cancer [40]. In addition, alterations in the gut microbiota have also been correlated with early-onset CRC [41,42].

The predominant phyla of gut microbiota are *Firmicutes* and *Bacteroidetes* [43]; however, in CRC, *Fusobacterium nucleatum* is the most prevalent gram-negative anaerobe gut bacterium [44] together with *Firmicutes* such as *Peptostreptococcus stomatis, Parvimonas micra,* and *Solobacterium moorei* [45]. A direct role of the microbiota in generating oncogenic mutations and promoting carcinogenesis has not been fully demonstrated; however, Clevers and coworkers recently showed that exposing human organoids to colibactin, a chemical that is synthetized by *E. coli*, caused a distinct mutational signature typically found in a sub-set of two human cancer cohorts and predominantly in CRC [46].

*Fusobacterium nucleatum* has been associated with poor clinical outcomes, MSI status, CpG island methylator phenotype (CIMP), and *BRAF* mutation status [47]. Notably, *Fusubacterium nucleatum* was negatively correlated with tumor-infiltrating lymphocytes in MSI tumors [48], and Guiney et al. observed an enrichment of *Fusobacteria* and *Bacteroidetes* in the most aggressive CMS1 (Consensus Molecular Subtype 1) CRCs, including all MSI CRCs [49]. These data suggest an association between gut microbiota and the MSI status of CRC; however, whether the MSI phenotype arises from a peculiar composition of microbiota has yet to be defined.

The spatial organization of microbiota within the gut may also influence gut microbiome dysbiosis. Dejea and colleagues demonstrated that polymicrobial bacterial biofilms were predominantly present in the right colon and in the normal mucosa adjacent to cancer cells. In addition, the presence of biofilm was associated with reduced E-cadherin expression, enhanced IL-6 and STAT-3 activation, and augmented crypt epithelial cell proliferation [50].

Interestingly, in different tumor types, the gut microbiota has been shown to impact clinical efficacy and patient response to CPIs. One study demonstrated that oral administration of *Bifidobacterium* and the anti-programmed death protein ligand 1 (anti-PD-L1) therapy abolished tumor growth, fostering dendritic cell function and CD8+ T cell priming in a melanoma pre-clinical model [51]. Concomitantly, in both a pre-clinical sarcoma model and in metastatic melanoma patients, the group of Zitvogel and coworkers revealed that the efficacy of anti-Cytotoxic T-Lymphocyte Antigen 4 (anti-CTLA-4) and T cell infiltration was dependent on the presence of *Bacteroides Thetaiotaomicron* or *Bacteroides fragilis* [52]. An elegant study confirmed these findings, demonstrating significant differences in the gut and oral microbiota of melanoma patients who responded to anti-programmed-death-1 (PD-1) [53]. In fact, they identified a favorable gut microbiota (enrichment in anabolic pathways) in responder patients as well as in germ-free mice that received a fecal transplant from responding patients. Other groups confirmed that the microbiota affects tumor response to CPIs, since the enrichment of *Faecalibacterium* and other *Firmicutes* was associated with beneficial clinical response to anti-CTLA-4 [54]. Finally, Peng and colleagues analyzed the response to anti-PD-1/anti-programmed-death-ligand 1 (anti-PD-L1) therapy in a cohort of gastrointestinal malignances, including CRC, and reported that a high *Prevotella/Bacteroides* ratio positively correlates with response to CPIs [55]. Of note, the role of microbiota has been studied not only in gastrointestinal cancers or in melanoma, but also in non-small cell lung cancer (NSCLC) and renal cell carcinoma (RCC), confirming that the occurrence of specific bacteria species may favor patient response to CPIs [56]. On the contrary, the microbiota may also interfere with tumor response to CPIs. For example, alterations in the composition of gut microbiota due to antibiotics were associated with primary resistance to CPIs in pre-clinical models (sarcoma and melanoma) [57] and in NSCLC and RCC patients treated with beta-lactam and quinolones [58].

All these data highlight a pivotal role of bacterial gut colonization in terms of immune system homeostasis and immunotherapy response across several cancer types. However, data about a potential interplay between the gut microbiota and CPIs in MSI CRC are still missing. Overall, therapeutic approaches aimed at modulating the microbiome could represent an opportunity to foster the efficacy of immunotherapy and overcome resistance to CPIs.

### 1.2. CRC MMR Deficiency and Immune Surveillance

The genetic differences between MSS and MSI tumors greatly affect the microenvironment landscape, the evolution of cancer in terms of progression, dissemination of the tumor cells, and also response to multimodality treatments reported in current clinical guidelines [59,60].

In 2016, a retrospective analysis of 1388 colorectal cancer tumors allowed for the classification of tumors in four consensus molecular sub-groups (CMS), based on immune cell compartments and fibroblastic and angiogenetic microenvironment [61]. The CMS1 sub-group was enriched for MSI tumors and expressed cytotoxic T cell markers. CMS2 was characterized by tumors with chromosomal instability and activation of the Wnt and Myc pathways. CMS3 was enriched in *KRAS* mutant tumors with disrupted metabolic pathways. Finally, CMS4 was the poor-prognosis mesenchymal sub-group with abundant infiltration of immune suppressive signatures. Interestingly, in both CMS1 and CMS4, the levels of CD8+ T and CD68+ cells (macrophages) were higher than in the other two sub-groups; however, the diversity in terms of suppressive function of macrophages in CMS1 and CMS4 remains largely unexplored.

**Figure 1:**
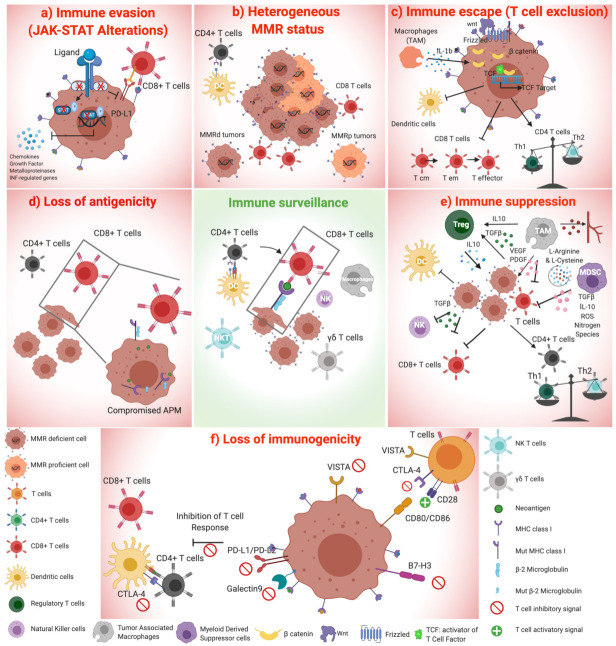
Genetic and non-genetic mechanisms of immune evasion in MSI tumors. MSI tumors may trigger a prolific immune response in the presence of CPIs, since they generate more tumor-specific antigens than MSS tumors, thereby inducing the immune surveillance (green panel). However, MSI tumors can deceive the immune system through several evasion patterns: (**a**) alterations in the JAK-STAT pathway compromise the immune response and negatively regulate PD-L1 expression; (**b**) MMR-deficient and -proficient neoplastic cells in the same tumor might impede the prolific response to CPIs; (**c**) the Wnt/β catenin pathway in MSI tumors contributes to T cell exclusion, favoring the immune escape; (**d**) the loss of antigenicity, due to the acquisition of mutations in specific genes (e.g., MHC class I, B2M), compromises the opportunity to present tumor-derived antigens to the immune system; (**e**), immune suppression exerted by Tregs, MDSC, and peculiar cytokines like TGF-β and IL-10 leads to an inhibitory activity towards the immune cells with cytotoxic properties allowing tumor proliferation, vascularization, and metastasis formation. T cm (central memory T), T em (effector memory T). (**f**) tumors may lose the ability to be immunogenic mainly by the expression of inhibitory immune checkpoints (e.g., PD-L1, VISTA) that induce T cell “anergy”; several checkpoint molecules are detectable on antigen presenting cells other than on the tumor cell surface such as PD-L1, PD-L2, CD80, CD86, Galectin 9, and B7-H3. In addition, VISTA is also present on T cells. Created with BioRender.com.

The contribution of the immune compartment in the evolution of CRC is well known, and the localization and phenotype of T-Cytotoxic, T-helper Type 1 (Th1), and T- memory infiltrating cells prominently affect survival of patients [62]. Since 1994, several authors highlighted the presence of robust T cell infiltration in MSI CRC tumors, which was later confirmed by several groups [23,63,64]. Accordingly, gene expression profiles of MSS and MSI tumors revealed an augmented expression of INF-ɣ in MSI specimens, supporting an active Th1 anti-tumoral response associated with MMR deficiency [65]. In 2006, Galon and colleagues underlined that the immune repertoire is a reliable and independent prognostic factor in CRC [62]. Then, in 2014, the same group defined the concept of “immunoscore” as a classification criterium based on the number and localization of CD3+ and CD8+ T cell subpopulations in the tumor microenvironment, independently from MMR-status-related classification [66]. Furthermore, in 2020, the immunoscore was included in the ESMO guidelines for the staging of CRC [67]. Relevantly, in stage I-III colorectal cancer, a high immunoscore is associated with a lower risk of relapse independently from MMR status [68,69], and similar data related to the importance of T- cell presence in tumor microenvironment have been reported for other cancer types [70,71]. Nevertheless, a conspicuous lymphocyte infiltrate is frequently associated with MSI status in CRC [72], suggesting that the better prognosis of MSI tumors is related to the high immune infiltration [69]. In addition, recent analyses performed with the CYBERSORT algorithm showed consistency with this data, observing an inflamed environment in MSI CRC tumors characterized by a prominent infiltration of M1 macrophages, CD8+ T cells, CD4+ cells, and natural killer (NK) cells [73]. Moreover, a conspicuous presence of NK cells in the CRC tumor microenvironment has been determined to be a positive prognostic factor [74]. All these data describe a dominant role of T cell response in CRC immune surveillance and highlight the need for new criteria to stratify patients that include the immune score to enlarge the cohort of CRC patients to be treated with CPI therapies [75]. While the status of the anti-tumoral adaptive immune response has been well described during the last few years, the knowledge about the contribution of innate immunity in CRC needs further elucidation. Some studies reported that significant dendritic cell (DC) infiltration is associated with a better clinical outcome and correlates with the infiltration of other immune populations [76,77]. This is expected and concordant with the fact that T cell activity strictly depends on antigen- presenting cells (APCs), which include DCs. Interestingly, Bauer and colleagues demonstrated in 2011 that S100+ dendritic cells are enriched in MSI CRC isolated from Lynch Syndrome patients [78]. Although a relevant portion of immune compartments infiltrate MSI tumors, around 30% of patients do not achieve any benefit from first-line CPIs [79]. In the next section, we will discuss the main mechanisms involved in immune escape despite the high level of antigenicity and immunogenicity of MSI colorectal cancers (Figure 1).

### 1.3. Mutational Characteristics of Mismatch-Repair-Deficient Cancer Cells

One key mechanism to maintain genomic integrity in cells is the mismatch repair (MMR) pathway. The MMR machinery consists of several multiprotein complexes capable of detecting and correcting insertions and deletions that occur during replication processes. MutL homolog 1 (MLH1), PMS1 homolog 2 (PMS2), MutS homolog 2 (MSH2), and MutS homolog 6 (MSH6) are the key players of the MMR system and work as heterodimers to guarantee the efficacy of the entire machinery [22]. Different genetic alterations can be inherited or occur spontaneously and lead to loss of MMR function, contributing to carcinogenesis and to the emergence of MSI tumors [80]. The majority of MMRd/MSI CRCs are caused by somatic mutations in MMR genes or epigenetic downregulation of *MLH1* expression [25]. However, 3% of MMRd/MSI CRCs arise in the context of hereditary non-polyposis colorectal cancer (HNPCC), also known as Lynch Syndrome, which is a hereditary cancer syndrome characterized by heterozygous germline mutations occurring in *MLH1*, *MSH2*, *MSH6*, or *PMS2* [81]. In addition, a minimal fraction of MSI patients develop tumors due to biallelic mismatch repair deficiency syndrome (BMMR-D), which is associated with early CRC onset [82]. Interestingly, while alterations in the MMR machinery are the “cause”, the compromised fidelity of nucleotide sequences is the “effect” that can be identified as a fingerprint on the DNA. Alexandrov and colleagues performed extensive work examining 4645 whole-genome and 19,184 exome sequences, identifying 49 single-base-substitution (SBS), 11 doublet-base-substitution, 4 clustered-base-substitution, and 17 small insertion–deletion signatures [83]. The mutational signatures of tumors were calculated using six substitution subtypes: C > A, C > G, C > T, T > A, T > C, and T > G, which generated 96 possible mutation patterns. The authors showed an enrichment in specific nucleotide changes (C > T and T > C) associated with MMRd tumors (SBS6, SBS15, SBS26 and SBS44 signatures) [83]. Doublet-base substitution (DBS) signatures specific to MMRd tumors were also identified (DBS7 and DBS10 signatures) as well as small insertion and deletion (ID) signatures (ID1, ID2, ID7 signatures) [83]. Importantly, multiple distinct mutational signatures may result from different mutational processes, as shown for MMRd and Polymerase Epsilon (*POLE)*/Polymerase Delta 1 (*POLD1)* mutant tumors (SBS14 and SBS20) [83,84,85]. Overall, the identification of mutational signatures in cancer is pivotal to understanding the biological process behind a cancer type, thus ultimately informing clinical decision making.

### 1.4. MMR Deficient Tumors Alert the Immune System, Triggering Cytotoxic T Cells and Inducing an INF-Mediated Immune Response

Among the main features of the MSI phenotype, an increased immune infiltration has been reported [65]. The findings in the recent years identified the prolific adaptive immune response as one of the main reasons why these tumors respond to CPIs [74]. However, while neoantigens’ role in triggering activated CD8+ T cells is largely known [86], recent publications have pinpointed the contribution of cytosolic DNA in the activation of INF-mediated immune response in MSI tumors [87].

#### 1.4.1. Neoantigen-Dependent Activation of Immune Surveillance in MMR-Deficient Colorectal Cancers

MMRd/MSI CRC tumors have been characterized for their antigenic properties, since they generate 10–50 times more tumor-specific antigens than MSS tumors [65,88]. A functionally compromised MMR machinery leads to the accumulation of single nucleotide variants (SNVs), insertions/deletions (indels), inversions, translocations, and other structural alterations that contribute to the mutational landscape of MMRd tumors [89]. All these alterations, if transcribed, translated, and presented by the MHC class I complex can generate new antigens (neoantigens) that, if recognized by the T cell receptor (TCR), may activate an immune response against cancer cells [90]. Notably, neoantigens are presented by MHC class I and II, triggering the activation of cytotoxic CD8+ T cells (MHC class I mediated) and the helper capacity of CD4+ T cells (MHC class II mediated) [91].

SNVs are individual nucleotide alterations that include synonymous changes (that do not affect the amino acidic sequence of the protein) and non-synonymous changes (that alter the protein sequence). The latter include non-sense and missense mutations that lead to a different amino acidic sequence compared to the wild-type protein.

These types of mutations are easy to identify using next-generation sequencing (NGS) technology. Conversely, small insertions and deletions generate frameshifts (FS), which are more challenging to detect [92]. Advanced bioinformatic tools can be used to identify and predict immune activating neoantigens by first identifying mutations or frameshifts in a specific genomic sequence followed by performing HLA-binding analyses using sophisticated software [93,94,95].

In 2017, we revealed that genetic inactivation of *MLH1* in pre-clinical models led to the dynamic accumulation of mutations that triggered a robust immune response [90]. Interestingly, we noted that the response was CD8+ T-cell-dependent, and the injection of MMRd (*MLH1* KO) tumor cell lines in mice triggered increased levels of TCR rearrangements in the blood as compared to MMR-proficient tumor cells. Additional studies have underlined the importance of neoantigens in triggering T cell infiltration and in positively affecting the response to immunotherapy in several tumor types [96,97,98].

The concept that the number of mutations correlates with the response to CPI has been elegantly addressed by Gubin and Schreiber who introduced the idea of “winning neoantigens” [99]. They were inspired by a study from Van Allen and collaborators whereby melanoma samples with high numbers of alterations had more chances to respond to immunotherapy due to increased odds of immunogenic neoantigens produced by tumor cells [100].

An additional key aspect to consider is the quality of alterations and how they can affect the immunogenicity of tumors. Specifically, single nucleotide changes may induce a significantly different number of neoantigens compared with frameshift mutations, most likely favoring neoantigens generated through indels. Even if a single immunogenic antigen can trigger an immune response, the number of putative neoantigens per alterations is higher if they arise from frameshifts. To test this hypothesis, the Swanton group analyzed a cohort of 5777 solid tumors across 19 cancer types from The Cancer Genome Atlas (TCGA), finding that two neoantigens could be produced from one frameshift generated by an insertion or deletion, whereas 0.64 neoantigens were achieved per SNV [80]. Interestingly, they noted that RCC patients had the highest number of indel mutations compared with other cancer types. Furthermore, they found CD8+ T cell signatures related to cytolytic activity in neoantigen-high RCC patients. Finally, they observed that the indel numbers were significantly associated with response to CPIs in melanoma patients. These findings demonstrated that indels generate a higher number of neoantigens than SNVs, thereby increasing the odds of neoantigen-associated immune activation and surveillance of tumor cells. Since every patient can have a peculiar mutational landscape, Leoni and collaborators analyzed 320 MSI tumor biopsies from TGCA, observing that 209 frameshift peptides were shared between patients [101]. In addition, considering an additional 20 MSI tumor patients, they identified 31 peptides in common with the initial cohort. Intriguingly, tumor specific neoepitopes derived from indel mutations have also been identified among patients with MSI endometrial, colorectal, and stomach cancers [102]. These findings pave the way to an “off-the shelf” vaccination strategy for treatment and prevention of MSI CRC tumors, although recent findings confirm that frameshift mutation frequency negatively correlates with the predicted immunogenicity due to the immune editing phenomenon [103].

The assumption that neoantigens derive from the coding region of the genome has been recently countered by the group of Perrault. Intriguingly, they proposed that in human and murine samples, almost 90% of peptides mounted on the MHC derive from non-canonical genomic sequences [104]. These findings are highly relevant in the neoantigen field and led to new hypotheses that a more extensive analysis of the non-coding portions of the genome could reveal several undefined features of MSI tumors and potentially lead to new mechanistic knowledge and help predicting the therapeutic outcome of patients.

#### 1.4.2. Cytosolic DNA Release Contributes to the Immunogenic Properties of MMRd Tumors

While the prevalent view is that the major contribution to the effectiveness of CPI in MMRd cancers is linked to the number of neoantigens, the observation that activation of the immune system can also occur through other pathways such as cGAS-STING (cyclic GMP-AMP synthase–stimulator of interferon gene) is gaining traction. Data suggest that in gastrointestinal diseases, cGAS-STING activation is key for the onset of colitis and CRC [105], while in other cancer types such as prostate, the accumulation of cytosolic DNA increases disease progression from non-malignant to hyperplasia to stage II [106]. Furthermore, STING activation triggers tumor growth in lung carcinoma pre-clinical models [107], most likely through interferon (INF)-mediated immune response, which has been shown to promote tumorigenesis. Finally, emerging pro-tumoral roles in metastatic processes have shown that cGAMP (cyclic guanosine monophosphate–adenosine monophosphate) can be transferred through gap junctions from tumor cells to astrocytes inducing interferon (IFN) and nuclear factor Kappa-ligand-chain-enhancer of activated B cell (Nf-kb) signaling and ultimately brain metastasis [108].

**Figure 2:**
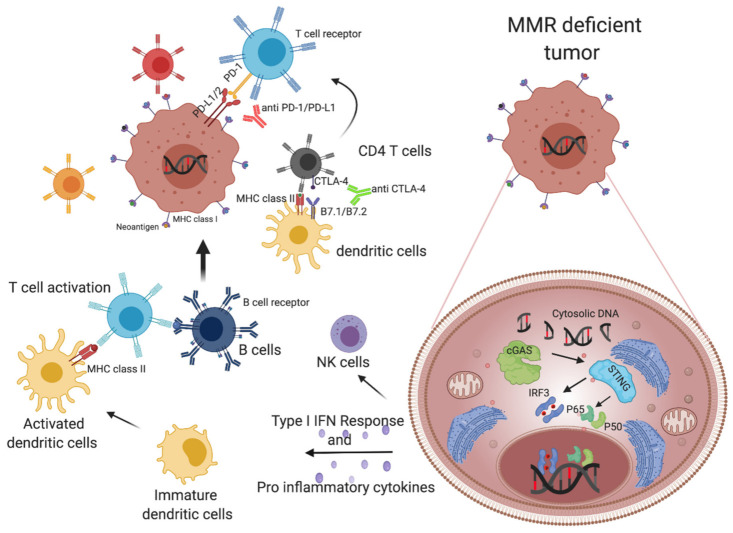
Cytosolic DNA and cGas-STING pathway activation lead to a strong immune response triggered by an antigen- and INF-mediated activation of adaptive immune compartments. The contribution of neoantigens to tumor regression of MSI tumors upon CPIs is a matter of fact. However, recent findings demonstrated that cytosolic DNA accumulation occurs in MSI cancer cells [87]. As consequence of this biochemical process, the cGAS-STING pathway is activated, resulting in the induction of type I INF mediated response and leading to the secretion of pro-inflammatory cytokines that sustain and foster anti-tumor response through multiple mechanisms. These findings lead to emergent strategies to trigger an immune response and to enroll patients with a positive predictive response to CPIs (PD-1/PD-L1 and CTLA-4). Created with BioRender.com.

Despite these data, several findings showed that triggering the cGAS-STING pathway regulates cellular senescence and apoptosis and enhances adaptive anti-cancer immunity [109]. Recently, the c-GAS-STING pathway was implicated in triggering immune response in an IFN-dependent manner (Figure 2) [110]. Specifically, activation of the immune system has been recently investigated, and studies have determined that DNA fragmentation induces INF response by STING, thus activating dendritic cell maturation and then CD8+ T cell activation [111]. Interestingly, stimulation of immune cells has also been described by trans-activation (tumor to immune cells). Particularly, cGAS expression by tumor cells triggers c-GAMP, which is translocated and activates STING and interferon-β production in myeloid and B cells [112,113]. Importantly, Woo and colleagues reported the presence of cytosolic tumor DNA in dendritic cells and macrophages in vivo. They showed that the activation of cGAS, STING, and interferon regulatory factor 3 (IRF3) was tumor-DNA-dependent and contributed positively to dendritic cell activation (Figure 2) [114]. Another immune mechanism involves the recruitment and activation of cytotoxic natural killer (NK) cells [115]. Notably, the DNA damage response in a lymphoma cell line led to STING-mediated induction of retinoic acid early transcript 1 ligand (RAE1). Then, RAE1 binding natural killer group 2 member D (NKG2D) that was expressed on the NK cells led the anti-tumoral immune response. In addition, STING activation both in tumor and immune cells may cooperate to produce different patterns of chemokines and thus induce tumor cytotoxicity by NK cells [116]. Interestingly, recent findings suggest that MMR deficiency and T-cell activation are linked by the cGAS-STING pathway [87]. Specifically, Lu and colleagues elegantly showed that in CRC and breast cancer models with defects in MMR, cytosolic DNA is accumulated and triggers a CD8+ T cell specific response. At the same time, Guan and colleagues disclosed that MLH1 regulates exonuclease 1 (EXO1) nuclease activity, and the impairment of the MLH1–EXO1 interaction leads to replication protein A (RPA) exhaustion and consequently DNA breaks and the release of nuclear DNA into the cytoplasm [117].

Overall, the cGAS-STING pathway is a promising therapeutic target in CRC. Indeed, exploiting cGAS-STING agonists could produce adjuvant effects and increase the efficacy of therapy such as radiation, vaccination, and immunotherapy [118]. These data highlight the contribution of cGAS-STING pathway, together with the MMRd-derived large number of neoantigens, to generating a productive immune response of MSI tumors once treated by immune-stimulating therapies (Figure 2).

### 1.5. The Role of Checkpoint Inhibitor Treatment in MSI mCRC Patients

The introduction of CPIs dramatically changed the algorithm of treatment for MSI mCRC. Initially, pembrolizumab was used as an advanced metastatic line of treatment and showed an impressive 40% objective response rate (ORR) with a 90% disease control rate (DCR) in MSI mCRC patients, compared to a 0% ORR and 11% DCR in patients with MSS tumors [16]. In the same setting, the combination of nivolumab plus ipilimumab achieved 55% ORR, 80% DCR, and 71% progression free survival (PFS) in 12 months [31].

The phase III randomized trial KEYNOTE-177 demonstrated the superiority of pembrolizumab over standard cytotoxic combinations +/− anti-epidermal growth factor receptor (EGFR) or anti-vascular endothelial growth factor (VEGF), the first line setting in MSI mCRC patients [79]. Notably, median progression-free survival (mPFS) in patients receiving pembrolizumab was 16.5 months versus 8.2 months among those who received cytotoxic agents (hazard ratio 0.60; 0.45–0.80) [79]. Furthermore, 83% of patients who responded to pembrolizumab were still responding at 24 months compared to only 35% of those treated with standard chemotherapy [79]. However, despite remarkable PFS and duration of response (DOR), 29.4% of patients exhibited primary resistance to pembrolizumab, compared to 12.3% of those enrolled in the standard treatment arm [79]. On the other hand, initial results from patients treated with the combination of nivolumab plus ipilimumab indicated that around 13% of MSI CRC patients exhibit primary resistance to therapy [119,120,121]. Further promising data from another phase III trial investigating the combination of nivolumab and ipilimumab (NCT04008030) in the same first-line setting are expected soon. In addition to the exploitation of CPIs in the metastatic setting, one trial testing the combination of short course nivolumab and ipilimumab in the neoadjuvant regimen in early-stage CRCs (NICHE trial) showed impressive pathological responses in both MMRd and MMRp CRC patients [122].

Primary and acquired resistance presently limit the efficacy of CPIs in MSI mCRC patients [16,31]; however, the mechanisms of resistance and immune escape to CPIs in this subset of mCRC remain unclear. To date, only MSI misdiagnosis has been suggested as a potential main mechanism of resistance to CPIs in the clinical setting [123,124].

## 2. Non-Genetic Mechanisms of Immune Evasion in Microsatellite Unstable CRC

### 2.1. Altered Expression of Inhibitory Immune Checkpoints

The expression of immune checkpoints is an immune evasion strategy intrinsic to tumor cells and is observed across different cancer types [125]. The expression of programmed cell death protein 1 (PD-1)/programmed cell death protein 1 ligand (PD-L1) axis, cytotoxic T-lymphocyte antigen 4 (CTLA4), lymphocyte activation gene 3 (LAG3), T-cell immunoglobulin, and mucin domain-3 (TIM3) proteins on tumor cells limits the activation of the adaptive antitumor response, ultimately associated to an “exhausted” immune system that is unable to perform prolific cancer killing (Figure 1) [126,127]. The high correlation between MMR deficiency and high tumor mutational burden (TMB) in CRC [128] suggests that the robust and continuous antigen presentation in MMRd tumors triggers immune over-activation, which results in the upregulation of immune checkpoint molecules as mechanisms of immune evasion. In 2018, a gene expression analysis revealed enrichment of immune checkpoints in tumor-infiltrating lymphocytes (TILs), in the invasive front and in tumor stroma of CRC MSI tumor compared to MSS tumor. In particular, the authors observed that CTLA-4 expression was statistically increased in all three compartments, LAG3 in TIL, and in the stroma, while the increase of PD-1 had no statistical power in all three compartments but was strongly higher in MSI specimens evaluated by immunohistochemistry [65]. Several studies also evaluated the association between MSI status and PD-L1 expression [65,129], and in one of these, PD-L1 positive cells were predominantly present in MSI tumors and not in MSS [65]. To study the distribution of PD-L1 expressing tumors among MSI, next-generation sequencing analysis of 11,348 samples across different cancer types revealed that among the small fraction of MSI (3%), only 26% of them expressed PD-L1 [130]. In addition, in another study, the evaluation of 4186 CRC cancer patients for TMB, PD-L1 expression and microsatellite status highlighted that in the cohort, only 12.8% were MSI, highly mutated, and PD-L1-expressing tumors [131]. Importantly, the evaluation of PD-L1 expression by immunohistochemistry could possibly be used to define a non-subjective biomarker of response. However, several technical variables such as the antibody, detection strategy, cutoff identified to define the positivity, tissue preparation, and staining of tumor and/or immune cells must be considered to define a PD-L1 positive tumor [132].

Other immune checkpoints are emerging in regulating cancer progression such as the V-domain Ig suppressor of T-cell activation (VISTA) expressed in CD4+ and CD8+ T cells but also in CD68+ cells (macrophages) [133]. The modulations of other immune checkpoints have been exploited in anti-tumoral strategies such as the inducible T-cell stimulator (ICOS) and OX-40, since it has been proven that ICOS agonist and anti-OX-40 based regulatory T cell (Treg) depletion sustains the anti-tumoral efficacy of CD4+ T effector cells [134]. Despite the well-known relevance of these immune checkpoint molecules, their contribution to MSI tumor progression has not yet fully addressed.

### 2.2. Cytokines, Chemokines, and Factors Orchestrate an Immune Suppressive Microenvironment in MSI Cancer

The immune suppressive environment is regulated by cross talk between myeloid and lymphoid cells and cytokines/chemokines and factors that cooperate to interrupt immune surveillance. The group of Bibeau performed an extensive analysis of 48 cytokines comparing MSS and MSI CRC patients [135]. MSI tumor samples displayed a peculiar cytokine profile compared to MSS tumors overexpressingchemokine (c-c motif) ligand (CCL-) 5, chemokine (C-X-C motif) ligand (CXCL-) -8, CXCL9, interleukin (IL-)-1β, CXCL10, IL-16, growth-regulated protein α (GROα), and IL-1 receptor agonist (IL1-ra). The differences were also maintained when considering MSS CRCs, which were highly infiltrated with CD3+ lymphocytes, excluding the GROα type. The authors also investigated the cytokine levels and specific immune cell density, finding that CXCL9 was associated with intra-tumoral intensity of CD3+ CD8+ cells in MSS tumors. The correlation between CXCL9 and T cells was higher in MSI and present in all tumor samples. In another study, the RNA analysis of 29 MSI and 104 MSS revealed higher expression of IL-18, IL-15, IL-8, IL-24, and IL-7 in MSI tumors [136]. Based on the literature, these chemokines can be produced by immune cells; however, there is evidence that CXCL1, CXCL10, and IL-8 can be produced by tumor and stromal cells [137,138,139]. The activator of transcription 3 (STAT3) is a key regulator of tumor-induced immune suppression [140]. As a matter of fact, the constitutive activation of STAT3 can be propagated through IL10, IL-8, and VEGF, from tumor to immune cells, which in turn leads to immune suppression [141].

One of the main players among immune suppressive cytokines is transforming growth factor β (TGF-β), a conserved cytokine produced by cancer cells, immune cells, and fibroblasts [142]. Interestingly, TGF-β can support cancer growth but also exerts its function directly on the immune system. In 1997, Alevizopoulos demonstrated that TGF-β suppressed the in vitro activation and proliferation of lymphocytes [143]. Currently, it is well known that the principal role of TGF-β signaling is in the control of inflammatory response, and several pioneering genetic experiments demonstrated that TGF-β is also required for the establishment and maintenance of T-cell tolerance [142,144,145]. In addition, in peripheral T cells, TGF-β restrains T-cell expansion and activity in response to exogenous stimuli [146,147]. TGF-β is key to instructing the regulatory program of T cells to promote differentiation of Treg and triggering Forkhead Box 3 (FOXP3) expression [148]. Furthermore, TGF-β blocks NK function at multiple levels [149,150] and suppresses the myeloid compartment [151,152,153]. The third exon of the TGF-β receptor 2 (TGFBR2) gene contains a 10-Adenine microsatellite sequence and is frequently mutated in the majority of MSI tumors; notwithstanding this, TGFβ signaling remains active in some tumors [154]. Furthermore, in a pre-clinical model of MSI CRC, restoring TGFβ signaling increases the metastatic rate of the tumor cell line [155]. Overall, despite the relevance of these pathways in MSS CMS4 CRC tumors and also in MSI CMS1 tumors, TGFβ remains a potential target to impede tumor growth.

### 2.3. cGas-STING Pathway May Alter the Immunogenicity of Cancer Cells and Favor an Immune Suppressive Microenvironment

The effects of cytosolic DNA and the role of the cGAS STING pathway in cancer progression has been previously described. Here, we will briefly discuss recent findings that emphasize the role of the cGAS-STING pathway in restricting the anti-tumoral immune response and favoring evasion from immune control. Recently, Li and colleagues demonstrated that the ectonucleotide pyrophosphatase/phosphodiesterase 1 (ENPP1) promotes metastasis by degrading cGAMP and contributing to the production of adenosine (an immune-suppressive and tumor-promoting metabolite) [156]. In human cancers, the expression of ENPP1 correlates with lower immune infiltration and resistance to CPIs [156]. Additional findings confirm that the cGAS-STING pathway may regulate the immunogenicity of cancer cells. Notably, cGAS-STING can directly activate the indoleamine 2,3-dioxygenase (IDO) on cancer cells. As a matter of fact, IDO deficient mice and specific inhibitors were capable of attenuating tumor growth in Lewis lung pre-clinical models [107]. Finally, high expression of STING was associated with increased regulatory cells and the immune suppressive IL-10 in human papilloma virus (HPV) positive tongue squamous cancers [157]. In line with these results, STING-deficient mice were prone to colitis-associated cancer, exhibiting low-levels of the tumor suppressor IL-22 binding protein [158]. Despite these indications, whether the cGAS-STING pathway might be exploited by MMRd tumors as a mechanism to evade immune control is currently unknown.

### 2.4. Suppressive Immune Cell Compartments in MSI CRC

The tumor microenvironment is often characterized by the infiltration of pro-tumoral immune populations that severely compromise antigen-reactive T cells activity [159]. An immune suppressive microenvironment is common in cancer to limit the control exerted by cytotoxic T cells, NK, and dendritic cells and to promote the immune escaping phase in cancer progression. Immune suppression can occasionally evolve into an extreme scenario such as the “immune-desert environment”, where the immune cells are strongly impeded from accessing the cancer niche [160,161,162]. In MSI tumors, the acquisition of low antigenicity and immunogenicity and the presence of immune-suppressive cell compartments such as myeloid-derived suppressor cells (MDSC), tumor-associated macrophages (TAM), or Tregs create a so-called “cold” tumor (Figure 1) [163].

#### 2.4.1. Regulatory T Cells

Regulatory T cells are usually defined by the T-cell subset expressing FOXP3+ marker and are crucial to maintaining physiological levels of activated immune response under normal conditions. In addition, they also play a role in restricting anti-cancer T-cell activity via several mechanisms such as secretion of inhibitory cytokines, cytolysis, metabolic disruption, and modulation of dendritic cell activity (Figure 1) [164,165]. Treg contribution to clinical outcomes of CRC is still unclear [166,167,168] but is likely due to the coexistence of an activated and a non-suppressive phenotype, as suggested by Picard and colleagues [74]. In some studies, the density of FOXP3+ tumor-infiltrating cells correlated positively with a prolonged patient survival [167,169], and accordingly, stage II CRC patients with poor CD3+ and FOXP3+ infiltration presented a higher risk of tumor progression [170]. On the contrary, Waniczek and collaborators observed that stromal enrichment of a subset of Treg cells was related to an increased risk of death and recurrence in colon cancer [166].

In 2008, Le Gouvello and coworkers described an increase in mRNA levels of FOXP3 in MSS tumors [171]. Concomitantly, Michel and colleagues performed a systematic evaluation of the FOXP3+ immune infiltration and MMR deficiency, revealing that MMRd/MSI-High (MSI-H)-deficient colorectal cancer displayed increased FOXP3-positive cells. These data suggested a possible contribution of Treg cells to the counterbalancing of the anticancer immune response armed against microsatellite unstable colorectal cancer [172]. Furthermore, Llosa and colleagues have elegantly observed a significant increase of FOXP3+ cells in MSI CRC, but no difference compared to the MSS counterpart was identified at the gene expression level [65]. Interestingly, two independent studies identified statistically significant enrichment of CD8- FOXP3+ cells in MSI tumors compared to MSS tumors, but this effect was only observed in the intra-tumoral compartments and not in the stromal area or in the tumoral invasive front [65,172]. These observations suggest that in a MSI tumor, the localization of immune cells might differentially affect the outcome of tumors and the response to therapies.

One fascinating observation derives from a reduction of mature CD208 dendritic cells concomitantly with an increased FOXP3+ density in MSI-H Lynch Syndrome tumors [78], suggesting that Treg components suppress and hinder the antigen presentation process mediated by mature dendritic cells and essential for cancer editing in MSI tumors.

Overall, the role of Tregs in the immune evasion of MSI CRC has not been completely elucidated. Several findings report the presence of Treg infiltration in MSI tumors, and the well-known suppressive potential of these cells prompts a possible involvement in immune evasion of MSI CRC tumor. Thus, Treg-depleting strategies [173] aimed to foster immune response are currently pursued to increase CRC responder patients to CPIs.

#### 2.4.2. The Role of Myeloid-Derived Suppressor Cells

Myeloid-derived suppressor cells are a heterogeneous group of cells that partially resemble neutrophils and monocyte phenotypes but that functionally present immune suppressive activity. The presence of MDSCs in the cancer niche is constantly pathological, and its contribution is described in several phases of cancer progression in CRC, from carcinogenesis to immune evasion (Figure 1) [174]. The interference of MDSCs with anti-tumor immunity occurs at several levels, involving innate and adaptive immunity and including a significant number of mechanisms directed to hamper T-cell activity [175,176]. The plethora of mechanisms by which MDSCs block both antigen-specific and non-antigen specific T-cell anti-tumor response include: (a) secretion of immune suppressive cytokines such as TGF-β and IL-10, (b) deprivation of metabolites essential for immune cell fitness and function such as L arginine and L-cysteine, (c) T-cell migration impairment, (d) interference with TCR structure and function throughout production of reactive oxygen species (ROS), and (e) CD4 T cells’ conversion toward regulatory suppressive phenotype [175]. Some studies revealed the presence of different subtypes of myeloid suppressor cells involved both in antigen-specific and non-specific T-cell response blocking in colorectal tumors [174]. Interestingly, a study revealed that Nifuroxazide treatment, a signal transducer and activator of transcription 3 (STAT3) inhibitor, was capable of reducing circulating and tumoral MDSC and exerting pro-apoptotic and anti-metastatic activity in the CT26 colorectal cancer mouse model [177]. Additionally, in a *KRAS* mutant CRC mouse model, suppression of the CXCL3-CXCR2 axis impaired MDSC infiltration, increase of adaptive immunity, and an unexpected efficacy of anti-PD-1 treatment [178].

In conclusion, different approaches have been designed to neutralize the MDSC suppressor activity such as MDSC depletion, inhibition of cell homing in tumor site and MSDC activity counteraction [179]. Despite this knowledge, the role of MDSC in MSI tumors needs further investigation to unveil the contribution in the immune surveillance and in the response to CPIs.

#### 2.4.3. Tumor Associated Macrophages

Macrophages are one of the more abundant components of the pro-inflammatory cancer microenvironment [180,181,182]. This innate immune population is crucial for the balance between anti-tumoral and pro-tumoral activity on the basis of phenotypic and functional features acquired in response to tumoral and environmental stimuli (Figure 1) [183]. Macrophages are considered extremely plastic, switching from an M1 anti-tumoral to a M2 pro-tumoral phenotype and vice versa [184]. For these concerns, the role of macrophages in CRC evolution is still highly debated, since many studies correlate tumor-associated macrophages’ (TAM) infiltration to anticancer activity and better clinical outcomes [185,186], while other observations link robust macrophage infiltration with disease fostering, progression, and immune evasion [187]. Interestingly, Naraynan and colleagues observed an increased M1 anti-tumor macrophages component in MSI compared with MSS tumors by RNAseq data and CIBERSORT algorithm, with a concomitantly augmented T-cell expression in both cytotoxic and helper compartments [186]. In contrast, a higher infiltration of CD163+ leukocytes, a marker of pro-tumoral M2 macrophages, has been identified in a cohort of Lynch Syndrome patients [78]. Accordingly, Hu and colleagues defined subtypes of MSI-H tumors from two different cohorts of patients: the MSI-H1 subgroup that was characterized by higher infiltration of M2 macrophages and a negative prognosis, and the MSI-H2 subgroup, featuring lower macrophage infiltration and better prognosis [188]. Recent data highlighted the contribution of macrophages in the response to CPIs. In particular, the compromised recruitment, differentiation, and survival of macrophages by administration of anti-colony stimulating factor 1 receptor (CSF1-R) in breast, pancreatic, and melanoma cancer models dramatically reduced intrinsic resistance to anti PD-1 therapy [189,190,191]. Accordingly, in 2017, it was reported that human and murine TAMs express PD-1, and this expression correlated with altered phagocytic function. More interestingly, in CRC human samples, the abundance of M2 PD-1+ macrophages augment with the disease stage [192], prompting the crucial involvement of TAM in anti-tumor response. The authors demonstrated that loss of the PD-1/PD-L1 axis of tumor macrophages triggers phagocytosis by inhibiting tumor growth in the CT26 murine colon cancer model [192].

These findings suggest promising therapeutic strategies that can be exploited to trigger a prolific adaptive immune response and impede the establishment of an immune-suppressive microenvironment such as pro-tumoral macrophages [193].

## 3. Genetic Mechanisms of Immune Evasion in MSI CRC

Genetic mechanisms of immune evasion are well characterized in MSI tumors. Exploiting an extensive molecular characterization on a large mixed cohort of CRC samples, Grasso and colleagues identified immune-related gene alterations enriched in MSI-high specimens [194]. To better validate the significance of these mutations against false positives due to the hypermutator phenotype of MSI-H specimens, the authors analyzed disruptive mutations, single-copy losses, and copy-neutral loss of heterozygosity (CN-LOH) in MSS and MSI-H tumors. The nature of the mutated genes revealed a strong selection for components of the antigen presenting machinery (APM) but also for genes involved in alternative mechanisms of immune evasion beyond the well-characterized adaptive T-cell response [194].

In 2012, a comprehensive molecular characterization, which included the mutational landscape, copy number alterations, and mRNA expression changes of 195 colorectal cancers, identified WNT as one of the main deregulated pathways in non- and hypermutated tumors [195]. In another cohort of CRC tumors, JAK1 indels were found in 20% of the 248 samples [196], while only 18% of the MSI CRC patients carried JAK1 indels [197]. Furthermore, recent findings highlight that common and rare germline genetic variants can shape the functional orientation of the tumor microenvironment [198]. Finally, recent findings are subverting the dogma that MMR status is mutually exclusive in the same tumor mass.

### 3.1. Antigen Presenting Machinery Disruption in MSI Tumors

Tumor antigen presentation plays a pivotal role in anti-cancer immunity, protecting the host from the development of neoplasia [199]. In MSI tumors, the contribution of tumor-derived antigens in the establishment of immune control is more relevant than in other tumor types. As a matter of fact, the altered mismatch repair machinery prompts the acquisitions of alterations that can be presented to the immune system as non-self-peptides [16,90,98,200].

The antigen presentation process is multifaceted and requires the involvement of several protein complexes. Briefly, exogenous and endogenous proteins are degraded by the proteasome while the heavy chain of classical MHC class I molecules is folded and dimerizes with Beta 2 microglobulin (B2M) in the endoplasmic reticulum. Calnexin prevents the aggregation of the heavy chain, which is then receptive for B2M. Next, calreticulin assists in associating the heavy chain–B2M to the peptide loading and assembly complex. This complex consists of the protein disulphide isomerase ERp57 (endoplasmic reticulum protein 57), Tapasin, and the Transporter Associated with antigen Processing (TAP1/2) proteins and is key for loading peptides on the MHC class I binding grove. Subsequently, the assembly complex dissociates from the MHC class I-B2M, allowing the passage from the endoplasmic reticulum to the Golgi and then to the plasma membrane [201]. In a cohort of 75 MSI primary tumors, Grasso and colleagues identified 27% of cases with biallelic disruption and 65% cases with minor damages to at least one of the genes essential for the antigen processing and presentation to CD8+ T cell. Overall, 57% of MSI cases display at least one mutation in human leukocyte antigen (HLA)-A, HLA-B, or HLA-C genes, including truncating mutations, mutations affecting HLA expression, and alterations that hinder correct interaction essential for the activation of T cells (Figure 1) [194].

In addition, NLR family card domain containing 5 (NLRC5) and regulatory factor X5 (RFX5), two regulators of the transcription of HLA genes [202,203], are mutated in a significant component of MSI-H samples analyzed, and these alterations lead to a reduction of the expression of HLA genes [194]. In another comprehensive analysis from the Dana Farber Cancer Institute (DFCI) database, 72% of MMRd CRC patients harbored alterations able to impair the APC. In particular, in 26% of patients NLRC5 mutations were associated with reduction of HLA expression [204]. These data confirm that HLA genes are key for anti-cancer immunity in MSI-H tumors, and alterations in the antigen-presenting mechanism are preferentially gathered in MSI tumors.

Other genes involved in the APC may be involved in modulating the antigenicity of MSI tumors.

Antigen peptide transporter-2 (TAP2), calnexin, tapasin, calreticulin, ERp57, and B2M genes encode for proteins that are essential for the maturation and the exposure of the antigen on the cell surface, and they appear to be mutated in a significant fraction of MSI tumors. Although alterations in TAP1/2 are less frequent than those in HLA, they are present in MSI cancers and can likely interfere with the antigen presenting process [205]. Finally, alterations in chaperone molecules such as Tapasin, Calreticulin, Calnexin, and ERp57 are present in MSI tumors; however, it is not completely understood whether the absence of these chaperons may fully compromise HLA assembly in the cells. To confirm this statement, findings from transgenic mice highlight how the HLA assembling is conserved despite the absence of calreticulin and tapasin [206].

A key regulator of APM is B2M, and, in 1996, Restifo and colleagues highlighted the relevance of this protein in HLA stability and antigen loading [207]. More recently, Zaretsky and colleagues showed that truncating alterations in B2M were one of the recurring findings in acquired resistance of melanoma to anti PD-1 [208]. Other findings support this evidence, confirming that patients with loss of heterozygosity of B2M show a negative prognosis if treated with anti PD-1 and anti CTLA-4 [209]. The same scenario was highlighted in patients with lung cancer treated with anti PD-1 [210]. Of note, in 2017, an extensive analysis of patients with MMRd tumors identified two of them who received anti PD-1 and developed progressive disease, and it was determined that both patients had acquired alterations in B2M [211]. On the contrary, recent data have shown that MMR-deficient colorectal cancer patients respond to anti PD-1 independently of the expression status of B2M proteins [212]. In our laboratory, we have also demonstrated that the genetic depletion of B2M impedes the immune surveillance on MMRd tumors; however, the absence of B2M does not preclude tumor regression upon CPIs [213]. In addition, we have clearly shown that tumor regression is driven by CD4+ T cells, since the peripheral depletion of this compartment completely abolishes the anti-tumoral effects of CPIs, while the efficacy was maintained despite CD8+ T cell depletion. Interestingly these findings were confirmed in B2M low-expressing MMRd tumor patients, whereby the response to anti PD-1 was associated with high number of tumor infiltrated CD4+ T cells.

These results highlight the relevance of the functional antigen-presenting machinery to the initiation of immune surveillance. However, at the same time, the peculiarity of MMRd tumors is largely confirmed, since a mechanism of secondary resistance to CPIs (B2M loss) in melanoma and lung cancer is confuted in MMRd tumors.

### 3.2. Deregulation of WNT Signalling Pathway Alters Tumor Microenvironment, Causing T-Cell Exclusion

Alterations occurring in the Wnt signaling pathway are common in cancer, and in CRC they occur in both MSI and MSS tumors [194]. Interestingly, analysis of the most mutated genes in a CRC cohort revealed that 13 out of 62 were related to the Wnt pathway [194]. The Wnt pathway is key in regulating the tumor microenvironment at several levels. In 2015, Spranger and colleagues elegantly demonstrated that the activation of the Wnt-β−catenin signaling pathway in melanoma caused the absence of T-cell-gene expression signature [214]. The same group showed that over expression of CTNNB1 (β-catenin) altered the T-cell-mediated control of melanoma [215]. The effect of APC loss acts throughout the β-catenin pathway immune suppressive activity on T-cell exclusion (Figure 1). In CRC, biallelic loss of APC determines a significant reduction of tumor infiltrating lymphocytes in both MSS and MSI-H CRCs, compared to those lacking biallelic APC disruptive mutations [194]. However, the incidence of APC alterations is higher in MSS compared to MSI CRC, and this may contribute to the higher immune control of MSI CRC [194]. Other contributions link the Wnt pathway with differentiation of CD4+ T cells, since β-catenin control the Th2 master transcription factor GATA binding protein 3 (GATA3) [216]. Finally, the role of the Wnt pathway in altering the T-cell-mediated anti-tumoral response has also been proven in other tumor types such as ovarian [217] and prostate cancers [218].

Studies have shown that the Wnt pathway may also regulate other immune compartments other than CD4+ and CD8+ T cells, such as in tumor latent competent cancers. These tumor sub-types consist of neoplastic cells with a limited outgrowth but with a high long-term survival and high cancer-initiating potential. Interestingly, in human breast and lung cancer cell lines, inhibition of the Wnt pathway caused the down modulation of NK cell ligands favoring the evasion from the immune surveillance in this tumor sub-type population [219]. The contribution of the Wnt pathway to immune evasion is exerted by modulating “marker of self” and “don’t eat me” molecules, such as CD47 [220], which impair the phagocytic activity of macrophages and dendritic cells [221]. Furthermore, PD-L1 expression is tightly regulated by the Wnt pathway in triple negative breast cancer [222]. Notably, Castagnoli and colleagues found that a Wnt agonist significantly increased PD-L1 at transcriptional and protein levels in three cell lines, while concordant results were obtained with XAV939 (Wnt inhibitor) [222].

Interestingly, if it is true that the Wnt pathway may alter the tumor immune microenvironment, the opposite might also be true. As a matter of fact, several findings confirmed how immune cells may regulate the Wnt pathway such as TAM that secrete interleukin-1 and phosphorylates glycogen synthase kinase 3 beta (GSK3b) and then increase the availability of β-catenin in colon cancer [223]. Furthermore, immune conditioned Wnt activation was also observed in skin cancer where MDSC augmented Wnt/β-catenin signaling via the secretion of Wnt ligand [224]. Hence, considering the relevance of this pathway in cancer progression, Grasso exploited the cohort of CRC patients to correlate the Wnt/β-catenin pathway and the adaptive immunity in the tumor microenvironment. Notably, he performed a complete characterization of T cells, demonstrating the inverse association between nuclear CTNNB1 expression and CD3+, CD8+, and CD45RO+ T cells. The biallelic loss of APC and, consequently, the upregulation of Wnt signaling occurred in 62% of MSS cases and 20% of MSI-H samples, thus indicating that T-cell infiltration was severely impaired independently of MSS and MSI status [194].

The Wnt pathway may clearly drive the immune response in cancer. However, it can also affect treatment response in several tumor types. Notably, the silencing of T-cell factor 4 (Tcf4) gene, a downstream effector of Wnt/β-catenin signaling, sensitizes colorectal cancer cell lines to oxaliplatin [225]. Furthermore, Wnt inhibitors have been shown to be effective in reducing the resistance of cancer cells to chemotherapeutic drugs. In HCT116 (an MSI CRC cell line), the acquisition of resistance to 5-Fluorouracil (5-FU) and oxaliplatin was associated with reduced Notch and Wnt signaling [226].

Finally, several inhibitors such as Esculetin, WNT5a inhibitor, and 6-bromo-indirubin-3′-oxime (GSK-3b inhibitor) may impact 5-FU resistance in tumor cell lines [226,227]. All of these findings confirm that alterations in the Wnt pathway contribute to sketching the immune repertoire of cancer, then favor the evasion from the immune control, and finally compromise the efficacy of chemo- and immune therapies.

### 3.3. Alterations in JAK-STAT Pathway Orchestrate Cancer Immune Evasion

Janus Kinases (JAKs) are members of a family of non-receptor tyrosine kinases with a key role in promoting tumor growth and regulating immune response. Other proteins, such as signal transducer and activator of transcription (STAT1/2), function downstream of JAK signaling and are pivotal for the regulation of chemokines, caspases, IFN-regulated genes, growth factors, and metalloproteinases (Figure 1) [228].

Data from TGCA sustain that MSI tumors (endometrial, colorectal, stomach, and prostate carcinoma) have recurrent frameshift mutations in JAK1, and these tumors showed reduced expression of interferon response signature [229]. In another study, thirty-seven patients with microsatellite unstable prostate cancer were analyzed, and JAK1 mutations were present in 68% of them. In addition, the authors compared these results with other tumors (endometrial, ovarian, colon, lung, and gastric adenocarcinoma) determining that there was a significant association between the tumor type and the percentage of JAK1 mutations [230].

Importantly, alterations in the JAK-STAT3 pathway have been identified as a mechanism of resistance to CPIs [231]. In 1998, a study showed that immune surveillance against cancer cells was affected by the interferon gamma pathway [232]. Recently, Ribas and colleagues extensively studied this aspect and proved several times that the exceptional response to anti PD-1 (75% ORR) of melanoma patients was severely compromised due to defects in the pathway involved in interferon-receptor signaling [208]. In addition, they also showed that the JAK-STAT pathway is a central regulator of PD-L1 and PD-L2 expression through IRF1 and STAT3 [233]. Other studies have emphasized the relevance of JAK1 and JAK2 mutations in the response to anti PD-1 of melanoma patients [208] and pre-clinical models have confirmed the evidence obtained from patients [234]. Although the prolonged exposure to IFN-γ drives the resistance to immune checkpoint blockade [235], the overall effect of INF-γ in cancer can be due to the anti-proliferative effects, the release of chemokines such as CXCL9 and CXCL10, or the regulation of HLA synthesis and expression [231].

### 3.4. Germline Genetic Variants Affect Different Immunomodulatory Pathways

One significant question that remains to be answered is how host genetic factors affect the immune system’s ability to elicit a response to a growing tumor. However, it has been reported that the abundance of cytotoxic T, NK, T follicular helper (Tfh) cells, and IFN signaling, associated with favorable prognosis and/or responsiveness to immunotherapy, can be hereditary in up to 15–20% of cases [236]. Interestingly, a recent seminal work on TCGA CRC samples described how the presence of germline MMR genes mutations led to higher immune infiltration only if the tumor displayed an MSI phenotype [198]. Further research is needed to determine if germline alterations other than MMR gene mutations are involved in influencing immune responsiveness in MSI mCRC patients.

### 3.5. The Intra-Tumoral Genetic Diversity of MMR Status as a Mechanism of Immune Escape

The definition of MMR status has been considered mutually exclusive in cancer; however, recent data highlight that MMRd and MMRp tumors can coexist in the same mass [11,237,238,239]. The identification of heterogeneous MMR status in the same tumor by immunohistochemistry is, in some cases, a consequence of technical caveats [240]; nevertheless, the presence of heterogeneous MMR-proficient and -deficient tumors has been recently identified in a fraction of CRC patients [240,241]. Molecular analyses performed on micro-dissected tumor areas demonstrated that both components are present in the same tumor, determining a mixed MMRp/MMRd tumor [237,241]. Intra-lesions’ heterogeneity was also observed in primary and metastatic CRC tumors in a study conducted on 369 patients. In most tumors, the MSS status was identified in both primary and metastatic specimens, whereas among 46 primary MSI tumors, nine of them were classified as MSS when the metastatic lesion was tested. Interestingly, the discrepancy was mainly limited to peritoneal and ovarian metastases [242]. If MMR heterogeneity is now confirmed, the implications of this phenomenon in cancer progression and response to therapies are still unclear and need to be thoroughly investigated. Loupakis and colleagues described a case of a mCRC patient harboring MMR heterogeneity at intra-tumor and inter-lesion level who experienced prolonged disease stabilization under nivolumab monotherapy and ipilimumab plus nivolumab treatment [241]. Surprisingly, the final progression of the disease was driven by the MMRd component [241]. In contrast, Kim and colleagues observed that a heterogeneous MLH1 positivity contributed to a lack of response to pembrolizumab in a metastatic MSI gastric cancer [11].

Summarizing, heterogeneous MMR patterns exist across different cancer types, and they inevitably could affect immune surveillance (Figure 1). Thus, the identification of tumors that are both MMRd and MMRp might subvert the dogma of the correct therapy for MSS and MSI CRC patients.

## 4. Translational Implications

A considerable number of MSI mCRC patients exhibit primary resistance to CPIs and another portion will develop secondary resistance mechanisms leading to disease progression [121]. Based on these observations, even if supporting data from translational studies are still lagging behind, several ongoing clinical trials are trying to bypass this limitation by exploiting combinations of CPIs with targeted agents (such as *KRAS* or *BRAF* inhibitors) or with cytotoxic agents and/or radiotherapy (Table 1). Apart from providing data on the efficacy of these combinations, these studies are also expected to provide translational data, which might be exploited to unveil potential biological mechanisms explaining resistance to CPIs in MSI mCRC.

Several inhibitory checkpoints have been identified [243], and the simultaneous administration of different inhibitory molecules might be promising to maximize the antitumor activity of immune system and refractoriness. The potential efficacy of this approach is also supported by evidence gathered in the advanced clinical setting where the combination of nivolumab (anti PD-1) and ipilimumab (anti CTLA-4) reached impressive DCR [31]. This combination in the upfront setting, based on phase II trial data [119], is expected to be more effective than pembrolizumab monotherapy. Interestingly, in the upfront setting, the efficacy of the nivolumab and ipilimumab combination is reported to be consistent regardless of *BRAF* and *RAS* status [120]. However, the safety of such a combination needs to be carefully evaluated. In this regard, the Check Mate 8HW trial (NCT04008030) is currently investigating this strategy and will soon provide more comprehensive data for the upfront treatment of MSI mCRC patients.

Targeting mutant *KRAS* has been a mirage for several years. However, recent important achievements in mCRC have shown that specific *KRAS^G12C^* inhibitors may severely affect tumor growth [244,245]. Interestingly, the use of AMG 510 (a KRAS ^G12C^ inhibitor) in pre-clinical models showed a prominent effect on anti-tumor immunity, causing an increase of CD3+, CD8+ T, and dendritic cell infiltration and inducing upregulation of MHC class I complex on tumor cells. In addition, the combination of anti-PD1 plus AMG510 in the same model has highlighted a synergistic effect, displaying a significantly higher efficacy compared to the single agent monotherapy and curing the majority of treated mice [244]. Moreover, in the KEYNOTE-177 trial, MSI *RAS* mutant patients did not achieve a survival benefit with CPIs when compared to patients treated with standard cytotoxic regimens. Overall, these results recommend a potential window for combining targeted agents and CPIs in the treatment of MSI CRC, potentially avoiding the use of cytotoxic agents in the first lines of therapy. Furthermore, given the prevalence of *BRAF* mutations among MSI CRC patients and the positive correlation between the expression of PD-L1, CD8+ tumor- infiltrating lymphocytes and *BRAF^V600E^* mutation [246], a further option of treatment is represented by the combination of CPIs and BRAF targeting agents or other agents targeting key players of the MAPK pathway [247]. In this regard, a number of clinical trials are currently ongoing to verify this hypothesis [247]. Interestingly, supporting the potential effectiveness of this strategy, initial results from combining dabrafenib, trametinib, and spartalizumab demonstrated a promising 35% ORR with a 75% DCR [248].

Differently from KRAS^G12C^ inhibitors and CPIs combinations, other trials are evaluating whether radiotherapy and chemotherapy can be exploited to enhance immunotherapy efficacy. As a matter of fact, therapeutic radiation has been identified as a modulating agent of the cancer immune environment, triggering presentation of cancer-specific antigens and contributing to an inflamed tumor microenvironment [249]. To this point, the combination of radiotherapy plus PD-1 inhibitor is now under investigation in MSI mCRC (NCT04001101, NCT03104439). In parallel, chemotherapy and anti-VEGF agents present several potential mechanisms to interact with immunological approaches, as reported in other malignancies such as NSCLC [250]. The multiple effects of chemotherapy on the immune system are emerging and involve the capacity to modulate the tumor microenvironment inducing antigen release, to foster the antigen presentation process throughout the increased expression of MHC class I and to impair the action of immune suppressive cell compartment [251]. This represents the background of the currently ongoing phase III COMMIT trial (NCT02997228). In addition, the contribution of chemotherapy might reduce the number of early progressors to CPIs [252]. To this end, chemo- and immune therapy approaches deserve validation in MSI mCRC patients.

In addition to these strategies, we envision that current preclinical data emerging on genetic and non-genetic mechanisms of resistance to CPIs might pave the way for the development of innovative translational approaches that boost CPI effectiveness and broaden the number of patients who will benefit from this therapeutic strategy. On this side, recent findings refute the dogma concerning MSS/MSI status. Indeed, these two genetic features are not mutually exclusive in the same tumor mass and can coexist [241]. Considering the clear distinction between the response of MSI tumors to CPIs, if compared to MSS, this statement might have a great impact on CRC treatment. Our laboratory recently demonstrated that temozolomide treatment, an alkylating agent approved in 2005 for first line therapy of glioblastoma [253], led to increased percentage of MMRd cells among a MC38 colorectal cell line population initially considered to be MMRp prior to treatment. Additionally, these selected cells were MLH1 KO, MSI, and able to provoke immune surveillance in mice [90]. These findings were also confirmed among a large cohort of CRC human cell lines. Furthermore, in agreement with in vitro observations, analysis of biopsies from eight patients relapsing upon temozolomide-based therapeutic regimens revealed O6-methylguanine-DNA methyltransferase (MGMT) re-expression (five patients) and MMR genes mutations (i.e., *MSH2* or *MSH6*) as main resistance mechanisms [90]. In both cell lines and biopsies, MMR inactivation led to increased mutational load and predicted neoantigens, suggesting an augmented immunogenicity. These preclinical data led to the clinical trial ARETHUSA (NCT03519412). Within ARETHUSA, MMR-proficient CRC patient tumors are tested for MGMT expression by immunohistochemistry, and those that are negative (e.g., for MGMT promoter methylation) are treated with temozolomide and then tested for tumor mutational burden in post-temozolomide tumor biopsies. Tumors with tumor mutational burden higher than 20 mutations per megabases undergo pembrolizumab treatment. This trial aims to test the hypothesis formed during preclinical studies that tumors with acquired resistance to temozolomide might be an MSI population and thereby will be sensitive to pembrolizumab treatment.

In conclusion, despite CPIs having revolutionized the landscape of treatment of MSI mCRC, new approaches combining different strategies are likely to boost the effectiveness of immunotherapy in this peculiar subset of patients. However, despite recent efforts, robust data from preclinical and translational studies as well as from early clinical trials are still lacking [121]. Different combinatorial approaches are currently under investigation (Table 1), and many others are likely to emerge based on preclinical elucidation of resistance mechanisms to CPIs.

## 5. Conclusions and Future Perspectives

The positive outcome of MSI mCRC upon CPI treatment is a matter of fact. However, the conspicuous fraction of patients who exhibits primary resistance or who develop acquired resistance during CPI treatments remains an unmet clinical need. High neoantigen burden characterizes MSI tumors and influences the mechanisms of immune escape, since most of them involve the compromised antigen-presenting machinery. Hence, mutations in MHC and in B2M are the most common in an MSI cohort, as extensively reviewed elsewhere [194]. However, recent data suggest that in addition to the compromised MHC class I–neoantigen complex as a mechanism of resistance, there exist a plethora of immune mechanisms that could be activated in the presence of CPIs [212,213]. In addition, new findings have emphasized the activation of the c-GAS-STING pathway in MSI tumors, demonstrating that the response to CPIs was INF-mediated. These data open the possibility of a new scenario where the regression of MSI tumors upon CPIs could be due to a combination of c-GAS STING pathway activation and neoantigens presented to immune cells. To elucidate whether alterations in the cGAS-STING pathway are part of the mechanisms of immune surveillance, further studies need to be conducted in MSI tumors. In this review, we also suggest that several other candidates deserve to be explored as drivers of immune evasion in MSI mCRCs. JAK-STAT pathway mutations are a mechanism of resistance to CPIs in cancer [231] and occur frequently in MSI tumors, limiting interferon gamma response signature expression [229]. WNT signaling upregulation has been observed in CRC patients, including MSI cases, and it is associated with immune infiltration impairment [194]. We also highlight how the microbiome and immune environment potentially have a crucial role in MSI tumor evolution under immune pressure and need to be deeply investigated. We have shed light on possible strategies to overcome the resistance to CPIs in MSI CRC, starting from the combinatorial approach of immune checkpoint inhibitors such as anti-PD-1 plus anti-CTLA-4 regimen. In addition, small molecules directed against common alterations (*KRAS* mut) trigger an inflammatory environment that might foster tumor immunogenicity and CPI-driven immune activation. Of note, cytotoxic agents and radiotherapy, the most common therapeutic strategies in cancer treatments to date, may act as inducers of the immunogenic environment, prompting an intriguing combination opportunity with CPIs (Table 1). However, we cannot exclude that other molecules other than alkylating agents might contribute to triggering a “hot” tumor microenvironment in MSI mCRCs. Our laboratory recently demonstrated that Vitamin C leads a prolific immune response against MSI tumors through the adaptive immune compartments of the host [254]. Whether the administration of Vitamin C could hamper the frequency of immune refractory MSI tumors needs to be further investigated. A key question remains to be addressed in the future regarding the full MSS/MSI status of tumor specimens. The identification of MSS tumor fractions in a MSI tumor leads to the identification of unconventional strategies to selectively target MSS tumor cells. Here, we have described the alkylating agents temozolomide and 6-Thioguanine and the clinical trial based on this hypothesis.

Overall, the findings reported herein highlight the properties of MSI CRCs, which have shown the most remarkable response to CPIs. The neoantigen burden and the contribution of cytosolic DNA are elements that might drive an effective immune mediated anti-tumoral response. However, despite these common properties, MSI tumors might be considered heterogeneous, since diverse immune-regulator profiles might govern the response to CPIs.

Hence, the identification of molecular mechanisms underlying MSI CRC, the role of the microenvironment, and finally the comprehension of the strategies to restore immune control are excellent ropes to precede the climb to the summit. To this aim, a strong collaboration between clinical and preclinical research to generate translational data is warranted to identify and overcome potential mechanisms leading to CPIs’ resistance in MSI CRCs.

## Figures and Tables

**Table 1:** Summary of potential clinical strategies with main ongoing and recruiting clinical trials aiming to avoid or reverse resistance to checkpoint inhibitors in metastatic colorectal cancer with particular regard to those with microsatellite instability (MSI).

Clinical Strategy	Trial Identifier	Phase	Regimen
CPIs combinations	NCT04008030 (CheckMate 8HW)	III	Nivolumab and ipilimumab vs. standard cytotoxic regimens
CPIs plus targeted agents - *RAS* inhibitors - *BRAF* inhibitors	NCT03785249 (Krystal 1)	I/II	MRTX849 and pembrolizumab
	NCT04185883 (Codebreak)	Ib/II	Sotorasib + PD1i
	NCT03668431	II	Dabrafenib + trametinib + spartalizumab
	NCT04294160	Ib	Dabrafenib + LTT462 (ERKi) + Spartalizumab (PDR001)
CPIs plus cytotoxic and/or anti-VEGF agents	NCT02997228 (COMMIT)	III	FOLFOX + bevacizumab + atezolizumab vs. atezolizumab monotherapy vs. FOLFOX + bevacizumab
CPIs plus radiotherapy	NCT04001101	II	Pembrolizumab ± RT
	NCT03104439	II	Nivolumab + ipilimumab + RT
Converting strategy	NCT03519412 (ARETHUSA)	II	Temozolomide followed by pembrolizumab

Legend: CPIs = checkpoint inhibitors; VEGF = vascular endothelial growth factor; vs. = versus; i = inhibitor.

## Data Availability

Not Applicable.

## Lexical of Abbreviations

5-FU	5-fluorouracil
APC	Antigen presenting cell
APM	Antigen presenting machinery
B2M	β 2 microglobulin
BMMR-D	Biallelic mismatch repair deficiency syndrome
CCL	Chemokine (cc motif) ligand
cGAMP	Cyclic guanosine monophosphate-adenosine monophosphate
c-GAS	Cyclic GMP-AMP synthase
CIMP	CpG island methylator phenotype
CMS	Consensus molecular subtype
CN-LOH	Copy-neutral loss of heterozygosity
CPIs	Checkpoint inhibitors
CRC	Colorectal cancer
CSF1-R	Colony stimulating factor 1 receptor
CTLA4	Cytotoxic T-lymphocyte antigen 4
CTNNB1	β -catenin
CXC	Chemokine (cxc motif)
CXCL	Chemokine (cxc motif) ligand
DBS	Double base substitution
DC	Dendritic cell
DCR	Disease control rate
DFCI	Dana Farber Cancer Institute
DOR	Duration of response
ENPP1	Ectonucleotide pyrophosphatase/phosphodiesterase 1
ERP57	Endoplasmic reticulum protein 57
EXO 1	Exonuclease 1
FOXP 3	Forkhead box 3
FS	Frameshift
GATA3	GATA binding protein 3
GROα	Growth regulated protein α
GSK3B	Glycogen synthase kinase 3 beta
HLA	Human leukocyte antigen
HNPCC	Hereditary non-polyposis colorectal cancer
HPV	Human Papilloma Virus
ICOS	Inducible T-cell costimulator
IDO	Indoleamine 2,3-dioxygenase
IL-	Interleukin-
IL1RA	IL-1 receptor agonist
IN/DEL	Insertion/deletion
INF	Interferon
IRF3	Interferon regulatory factor 3
JAK	Janus kinases
LAG3	Lymphocyte activation gene 3
M1	M1-like macrophages
M2	M2-like macrophages
mCRC	Metastatic CRC
MDSC	Myeloid derived suppressor cells
MGMT	O6-methylguanine-DNA methyltransferase
MHC	Major histocompatibility complex
MLH1	MutL homolog 1
MMR	Mismatch repair
MMRd	Mismatch repair deficient
MMRp	Mismatch repair proficient
mPFS	Median progression free survival
MSH2	MutS homolog 2
MSH6	MutS homolog 6
MSI	Microsatellite instability
MSS	Microsatellite stable
NFKB	Nuclear factor Kappa-ligand-chain-enhancer of activated B cells
NGS	Next generation sequencing
NK	Natural killer
NKG2D	Natural killer group 2 member D
NLRC5	NLR family CARD domain containing 5
NSCLC	Non-small cell lung cancer
ORR	Objective response rate
OS	Overall survival
PD-1	Programmed cell death protein 1
PD-L1	Programmed cell death protein ligand 1
PFS	Progression free survival
PMS2	PMS1 homolog 2
POLD1	Polymerase delta 1
POLE	Polymerase epsilon
RAE1	Retinoic acid early transcript 1 ligand
RCC	Renal cell carcinoma
RFX5	Regulatory factor X5
ROS	Reactive oxygen species
RPA	Replication protein A
SBS	Single base substitution
SNVs	Single nucleotide variants
STAT	Signal transducer and activator of transcription
STING	Stimulator of interferon gene
TAM	Tumor associated macrophages
TAP	Transporter associated with antigen processing
TCF4	T cell factor 4
TCGA	The Cancer Genome Atlas
TCR	T cell receptor
Tfh	T follicular helper
TGFBR2	TGF-β receptor 2
TGF-β	Transforming growth factor β
Th1	T-helper type 1
TIM3	T-cell immunoglobulin and mucin domain-3
TMB	Tumor mutational burden
Treg	Regulatory T cells
VEGF	Vascular endothelial growth factor
VISTA	V-domain Ig suppressor of T cell activation
